# Metabolic Health, Obesity, and Renal Function: 2013–2018 National Health and Nutrition Examination Surveys

**DOI:** 10.3390/life11090888

**Published:** 2021-08-28

**Authors:** Kathleen E. Adair, Rodney G. Bowden, LesLee K. Funderburk, Jeffrey S. Forsse, Kelly R. Ylitalo

**Affiliations:** 1 Department of Health, Human Performance, and Recreation, Robbins College of Health and Human Sciences, Baylor University, One Bear Place #97313, Waco, TX 76798, USA; leslee_funderburk@baylor.edu (L.K.F.); jeff_forsse@baylor.edu (J.S.F.); 2 Department of Public Health, Robbins College of Health and Human Sciences, Baylor University, One Bear Place #97343, Waco, TX 76798, USA; rodney_bowden@baylor.edu

**Keywords:** chronic kidney disease, CKD, metabolic phenotypes, obesity, metabolic risk factors

## Abstract

Rising rates of metabolic syndrome, obesity, and mortality from chronic kidney disease (CKD) have prompted further investigation into the association between metabolic phenotypes and CKD. Purpose: To report the frequency of strictly defined metabolic phenotypes, renal function within each phenotype, and individual risk factors associated with reduced renal function. We utilized the 2013–2018 National Health and Nutrition Examination Surveys (NHANES) and complex survey sample weighting techniques to represent 220 million non-institutionalized U.S. civilians. Metabolic health was defined as having zero of the risk factors defined by the National Cholesterol Education Program with the exception of obesity, which was defined as BMI ≥ 30 kg/m^2^ in non-Asians and BMI ≥ 25 kg/m^2^ in Asians. The metabolically healthy normal (MUN) phenotype comprised the highest proportion of the population (38.40%), whereas the metabolically healthy obese (MHO) was the smallest (5.59%). Compared to the MHN reference group, renal function was lowest in the strictly defined MUN (*B* = −9.60, *p* < 0.001) and highest in the MHO (*B* = 2.50, *p* > 0.05), and this persisted when an increased number of risk factors were used to define metabolic syndrome. Systolic blood pressure had the strongest correlation with overall eGFR (*r* = −0.25, *p* < 0.001), and individuals with low HDL had higher renal function compared to the overall sample. The MUN phenotype had the greatest association with poor renal function. While the MHO had higher renal function, this may be due to a transient state caused by renal hyperfiltration. Further research should be done to investigate the association between dyslipidemia and CKD.

## 1. Introduction

In the past three decades the incidence of end-stage renal disease (ESRD) has increased by approximately 93% [1], and chronic kidney disease (CKD) is the third fastest growing cause of premature mortality [2]. CKD is a costly [3] and burdensome health issue that more often results in premature mortality than in ESRD [4]. Steady increases in rates of metabolic syndrome and obesity are occurring in the U.S., with both conditions recently exceeding previous levels at 34.2% [5] and 42.6% [6] of the U.S. population, respectively. Approximately 15% of U.S. adults are estimated to have CKD [7], and it is likely that the prevalence will increase given the associations of CKD with metabolic risk factors, such as type 2 diabetes mellitus (T2D), hypertension (HTN) [7], and obesity [7,8,9].

Metabolic phenotypes, which take into account metabolic risk factors and obesity, have been utilized to assess the risk of various outcomes, such as cardiovascular disease (CVD), mortality, and CKD. A recent meta-analysis by Alizadeh et al. [10] analyzed nine prospective cohort studies that compared CKD risk among metabolic phenotypes and found that the metabolically healthy obese (MHO) and the metabolically unhealthy normal weight (MUN), termed the “intriguing” phenotypes, shared a similarly elevated risk of developing CKD, with pooled relative risks (RR) of 1.55 and 1.58, respectively. This meta-analysis included studies with primarily Asian populations, limiting generalizability, and the definitions of the metabolic phenotypes varied, hindering the comparability between studies.

Prior research in the area of metabolic phenotyping has reported equivocal findings regarding the number of risk factors used to define the “metabolically unhealthy” status, with the most strict definitions determining that one or more [11,12,13,14] risk factors should be considered unhealthy and the more liberal ranging from two or more [15,16] to three or more [17,18,19,20] risk factors. A recent publication by Lavie et al. [21] proposed a harmonized definition that classifies the metabolically healthy phenotype as having zero of the four metabolic risk factors. This rationale is based on the notion that individuals with hyperglycemia, dyslipidemia, and/or hypertension cannot be considered “healthy” and therefore should not be classified as such [21]. Several large studies [13,14,22] have previously used this definition, and several more [11,12,23] have adopted it since it was first proposed by Lavie et al.

In this study, our primary purpose was to report the prevalence of the strict metabolic phenotypes in the U.S. population utilizing NHANES data and complex survey sample weighting. Additionally, we reported the association between renal function and the metabolic phenotypes, utilizing the three most common definitions of metabolic health. Lastly, we identified and reported the individual risk factors associated with reduced renal function.

## 2. Materials and Methods

The institutional review board at Baylor University determined the present study exempt from review [IRB ID# 1505514-1]. The project was classified as non-human subjects research because the data are deidentified and widely available for use via the CDC. Survey sample weighting, which includes a complex, four-stage, probability cluster, was utilized for the present analyses. Sample weighting procedures are outlined by the National Center for Health Statistics Estimating and Weighting Procedures documents [24,25].

### 2.1. Study Sample

The National Health and Nutrition Examination Surveys (NHANES) are studies conducted in 2-year cycles by the Centers for Disease Control and Prevention (CDC). The NHANES design utilizes complex survey sample weighting procedures to produce nationally representative health statistics for the U.S. The population sampled by NHANES was limited to civilian, non-institutionalized individuals who lived within the U.S. at the time of sampling. In order to increase the reliability and precision of weighted estimates for underrepresented populations, oversampling of individuals 60 and over, African Americans, Asians, and Hispanics was routinely conducted. Sample weights were assigned to each individual in a sample in order to extrapolate the results to a represent all non-institutionalized U.S. civilians.

The inclusion criteria for the study required subjects to have completed one of the three most recently published cycles of the NHANES survey, which included the 2013–2014, 2015–2016, and 2017–2018 cycles. Inclusion criteria further required that subjects be between the ages of 18 and 79 and have complete study information to classify metabolic and renal health. The upper age limit was chosen because individuals 80 years and older in the NHANES dataset are top coded at 80 for subject deidentification; therefore, age cannot be controlled for over 79 years. The biological markers used to identify metabolic and renal health included fasting glucose, fasting triglycerides, high-density lipoprotein (HDL), blood pressure, body mass index (BMI), age, sex, race, and serum creatinine (SCr) value. Subjects were excluded from the study if they reported pregnancy and/or tested positive for a pregnancy test. Additionally, individuals who reported being on dialysis in the 12 months prior to the study were excluded from analysis.

The initial study sample included 29,400 subjects. A total of 12,594 did not meet the inclusion criteria for age, 190 were pregnant at the time of the study, 9988 did not have biological markers sufficient to classify metabolic and/or renal health, and 18 subjects reported use of dialysis. The final sample that was analyzed included 6610 U.S. citizens, which was representative of a population size of 220,388,819 individuals after the NHANES survey sample weights were applied.

### 2.2. Definition of Metabolic Phenotypes

Metabolic risk factors were defined using criteria from the National Cholesterol Education Program’s (NCEP) Adult Treatment Panel III (ATP III) [26], with the exception of obesity, which was defined as a BMI > 30 kg/m^2^ for all non-Asian individuals and a BMI > 25 kg/m^2^ for all individuals identified as Asian [16,18,20]. Metabolically healthy or unhealthy status was determined by the four remaining metabolic risk factors: hyperglycemia, which was defined as a fasting glucose ≥100 mg/dL or prescription medication for hyperglycemia; the two dyslipidemia criteria, which were defined as a fasting triglyceride ≥150 mg/dL, a high-density lipoprotein level <40 mg/dL for males, <50 mg/dL for females, or a prescription medication for dyslipidemia; and hypertension was defined as a resting systolic blood pressure >130 mmHg, a resting diastolic blood pressure >85 mmHg, or prescription medication for hypertension (Table 1). In the primary analyses, metabolic health was defined as the absence of all metabolic risk factors in Table 1, excluding the measure of obesity. Therefore, the metabolically healthy normal weight (MHN) phenotype was defined as the absence of all metabolic risk factors and absence of obesity; metabolically healthy obese (MHO) required the absence of all metabolic risk factors and presence of obesity; metabolically unhealthy normal weight (MUN) required the presence of one or more metabolic risk factors and absence of obesity; and the metabolically unhealthy obese (MUO) required the presence of one or more metabolic risk factors and presence of obesity.

### 2.3. Renal Outcome Measures

Renal function was calculated using the Chronic Kidney Disease Epidemiology Collaboration (CKD-EPI) equation [27]:

$$\mathrm{eGFR}=141\times \min{\left(\frac{SCr}{\kappa },\;1\right)}^{\alpha }\times \max{\left(\frac{SCr}{\kappa },1\right)}^{-1.209}\times 0.993^{Age}\times 1.018\;\left(if\;female\right)\times 1.159\;\left(if\;Black\right)$$

where eGFR is the estimated glomerular filtration rate, *SCr* is serum creatinine collected as part of the standard biochemistry profile using the DxC 800 chemistry analyzer, κ is 0.7 if female or 0.9 if male, α is −0.329 if female or −0.411 if male, min is the minimum of $\frac{SCr}{\kappa }$ or 1, and max is the maximum of $\frac{SCr}{\kappa }$ or 1. The CKD-EPI equation has been reported to be more accurate than the MDRD equation in individuals with higher GFRs [27]. CKD was defined as an eGFR < 60 mL/min/1.73 m^2^ (categories G3 to G5) and/or an albumin to creatinine ratio ≥30 mg/g [28]. All individuals who reported use of dialysis in the 12 months prior to the study were excluded from the analyses.

### 2.4. Questionnaires, Examinations, and Laboratory Data

The NHANES interview-style questionnaires include demographic, socioeconomic, dietary, health history, and lifestyle information. Age, binary sex, and race/ethnicity were determined by questionnaires that were asked in the home by trained interviewers using the Computer-Assisted Personal Interview (CAPI) system. Total caloric intake was determined using two 24-h dietary interviews, and a composite variable was created to average dietary intake for two-day samples. Dietary intakes were assessed on all days of the week, with the 2 measurements typically separated by 3 days. Eighteen percent of the dietary intake information was missing in the present sample. Subsample weights (WTDR2D sample weight variable) were utilized to marginally adjust for race and Hispanic origin, age group, sex, weekday-weekend categories, and day-two non-responders. SES was determined by dividing family (or individual) income by the poverty guidelines defined by the U.S. federal government. Subjects who fell at or below 100% of the poverty level for the given year, which is a common criterion for determining eligibility in federal assistance programs [29], were considered low SES. Physical activity (PA), reported in minutes per day and number of days per week, was classified using the guidelines from the PA Guidelines Advisory Committee Report [30]. Individuals were considered physically active if they took part in ≥150 min of moderate-intensity recreational PA per week, ≥75 min of vigorous-intensity recreational PA per week, or an equivalent combination of the two [30,31]. Implausible PA values were reported in this sample; therefore, values ≥4 h per day of recreational PA were top-coded at 4 h. There was 49% missingness in the PA variable. Subjects were considered smokers if they have smoked at least 100 cigarettes in their lifetime or if they reported having smoked in the past 5 days. All others were considered non-smokers. International Classification of Diseases, Tenth Revision (ICD-10) codes were used to determine prescription medication (Rx) information for hyperglycemia (R73, E11, E11.2, E11.2P, E11.4, and E11.P), hypercholesterolemia (E78.0, E78.0P, and E78.1), and hypertension (I10 and I10.P).

The NHANES examination includes anthropometric measures, blood pressure, blood panels, and urinalysis. BMI was calculated using height, which is measured in meters (m) on a calibrated stadiometer; and weight, which is measured on a calibrated digital weight scale or a portable scale. The waist circumference (WC) was taken at the level of the uppermost lateral border of the iliac crest and reported in centimeters (cm) for each subject. Three consecutive measures of blood pressure (BP) are taken after a 5-min seated rest period. In cases where the BP measurement was interrupted or incomplete, a fourth measure was taken and reported. The present analysis reported the mean blood pressure for each subject by averaging the three available systolic and diastolic blood pressures. Fasting blood samples were taken and reported for blood lipids and blood glucose. The lipid sample was analyzed using the Roche/Hitachi Cobas 6000 analyzer (Roche Diagnostics, Indianapolis, IN, USA), and the serum low-density lipoprotein (LDL), expressed in milligrams per deciliter (mg/dL) was calculated utilizing the Friedewald calculation [32]. Fasting plasma glucose was analyzed using the Roche Cobas C311 system. Serum high-sensitivity C-reactive protein (hs-CRP) levels were measured beginning in the 2015–2016 cycle of NHANES; therefore, 36.7% of the sample has missing values for this variable since it was not collected in the 2013–2014 cycle. The Beckman UniCel^®^ DxC 600 and 600i Synchron chemistry analyzers (Beckman Coulter, Brea, CA, USA) were used to measure hs-CRP in the 2015–2016 cycle, and the Roche Cobas 6000 was used in the 2017–2018 cycles. The homeostatic model assessment of insulin resistance (HOMA-IR), which is a method utilized to quantify insulin resistance and beta-cell function, was calculated using the following equation [33]: fasting glucose (mmol/L) × fasting insulin (microU/mL)/22.5. The albumin to creatinine ratio was reported in mg/g utilizing the fluorescein immunoassay by Sequoia-Turner Digital Fluorometer, Model 450 (Sequoia-Turner Corporation, Mountain View, CA, USA) to determine urinary albumin, and the Roche Cobas 6000 Analyzer was used to measure urinary creatinine.

The percentage of glycated hemoglobin (HbA1c) was not reported in the present study because its value was determined by questionnaire rather than a blood panel. Alcohol intake was not analyzed because the reporting method changed during the 2017–2018 cycle and could not be compared to prior surveys.

### 2.5. Statistical Analysis

Statistical analyses were conducted using SAS version 9.4 (SAS Institute Inc., Cary, NC, USA). A DOMAIN statement was used to analyze the subpopulation meeting study inclusion criteria. Masked variance pseudo-primary sampling unit (PSU), masked variance pseudo-stratum, and fasting subsample 2-year mobile examination center (MEC) weights from NHANES were used for sample weighting. Unweighted demographic information was represented using means and standard deviations (SD) for continuous variables or frequencies and percentages (n, %) for categorical variables. Weighted demographic data were reported for the total sample and metabolic phenotypes using a weighted mean and standard error of the mean (SE) for continuous variables or a percentage (%) and the standard error of percent (SE) for categorical variables. Simple regression analyses of weighted data were used to identify statistical differences between continuous demographic variables. Chi-square (χ^2^) tests were used to identify statistical differences between categorical demographic variables. Pearson’s product moment correlation coefficients (*r*) were used to identify correlations between two continuous variables. Linear regression models with complex survey sample weighting were used to determine the influence of metabolic phenotype on renal function. In model 1, we considered one metabolic risk factor to be unhealthy; in Model 2, we considered 2 risk factors to be unhealthy, and in Model 3, we considered 3 risk factors to be unhealthy. For all analyses, the level of significance was set a priori at α = 0.05.

## 3. Results

The weighted sample population of 6610 subjects who met the study inclusion criteria represented 220,388,819 non-institutionalized U.S. civilians. The weighted and unweighted demographic data are represented in Table 2. The prevalence of obesity was 42.49%, with an average BMI of 29.4 (SE = 0.18). The prevalence of individuals with at least one metabolic risk factor (excluding obesity) was 75.30%, and only 19.11% of the sample was metabolically healthy and non-obese. The most frequent metabolic phenotype was the MUN phenotype (38.40%) followed by the MUO (36.90%), and the phenotype that represented the smallest proportion of the sample was the MHO (5.59%). The metabolically unhealthy phenotypes were more likely to be male, older age, current or former smokers, have metabolic risk factors, and have poor renal function, whereas the metabolically healthy individuals tended to have higher HDL-cholesterol and reported that they engaged in greater amounts of recreational physical activity. The obese phenotypes were more likely to be female, non-Hispanic (NH) Black Americans, and have higher levels of hs-CRP, whereas the normal-weight individuals were more likely to be NH White or NH Asian despite more conservative obesity cutoff values for NH Asians. There was no statistically significant difference between phenotypes for daily caloric intake or frequency of individuals with low SES.

The linear regression analyses in Table 3 utilized three consecutive models to demonstrate eGFR in the metabolic phenotypes ranging from a strict definition of metabolic health to the conventional definition outlined by the NCEP ATP III. The most conservative definition defined metabolic health as 0 risk factors with the exception of obesity, where the frequency of MHN was 19.11%, MHO was 5.59%, MUN was 38.40%, and MUO was 36.90%. When metabolic health was defined as one or fewer metabolic risk factors, the frequency of each phenotype shifted towards metabolically healthy: MHN accounted for 36.67% of the population, MHO was 15.56%, MUN was 20.85%, and MUO was 26.92%. Further shifts towards the metabolically healthy phenotypes were demonstrated when metabolic health was defined as two or fewer metabolic risk factors: MHN accounted for 48.64% of the population, MHO was 27.36%, MUN was 8.88%, and MUO was 15.12%. Across models, the eGFR in the MHO phenotype was slightly higher than that of the reference although this association was not found to be significantly different. The MUN and MUO phenotypes had significantly lower eGFR than the reference group (MHN). Across all three models, the MUN phenotype consistently demonstrated the lowest average eGFR compared to all other phenotypes. This finding is consistent with the demographic information represented in Table 2.

Correlates of eGFR and SCr are demonstrated in Table 4. The risk factors found to be most closely associated with low renal function were systolic blood pressure (eGFR, *r* = −0.250, *p* < 0.01, SCr *r* = 0.105, *p* < 0.001) and waist circumference (eGFR, *r* = −0.175, *p* < 0.01, SCr, *r* = 0.096, *p* < 0.001). HDL demonstrated a significant negative association with SCr (*r* = −0.123, *p* < 0.001), indicating that as HDL increases, SCr decreases. In the MHO phenotype, which included individuals with no risk factors except obesity, the fasting triglyceride levels had a small inverse relationship with eGFR (*r* = −0.159, *p* < 0.05), and HDL, BMI, and waist circumference demonstrated small negative relationships with SCr (*r* = −0.172, −0.165, and −0.123, respectively, *p* < 0.05 for all). Renal function in the MUN phenotype demonstrated significant correlations with systolic blood pressure (eGFR, *r* = −0.269, *p* < 0.01, SCR, *r* = 0.106, *p* < 0.001), BMI (eGFR, *r* = −0.124, *p* < 0.001, SCR, *r* = 0.061, *p* < 0.05), and waist circumference (eGFR, *r* = −0.282, *p* < 0.001, SCr, *r* = 0.187, *p* < 0.001). In the MUN group, eGFR and SCr were negatively correlated to HDL (eGFR, *r* = −0.088, *p* < 0.001, SCr, *r* = −0.126, *p* < 0.01).

**Figure 1:**
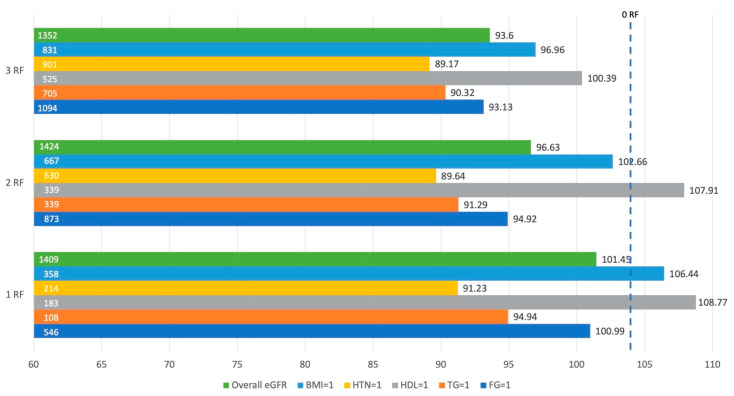
eGFR and Metabolic Risk Factors.

Figure 1 demonstrates the average eGFR in individuals with one, two, or three risk factors. This figure represents the impact of each metabolic risk factor, including obesity, on eGFR. The reference point was an individual with 0 risk factors (eGFR = 103.93 mL/min/1.73 m^2^). Regardless of the number of risk factors an individual had, those with hypertension consistently had the lowest eGFR, and the eGFR in those with hypertension decreased as the number of risk factors increased. Dyslipidemia in the form of high fasting triglycerides was the second most detrimental risk factor associated with eGFR. Individuals with low HDL as defined by the NCEP ATP III criteria consistently demonstrated the highest eGFR despite this being a metabolic risk factor.

This figure represents the average estimated glomerular filtration rate (eGFR) in individuals with one, two, and three risk factors, including obesity. By highlighting each individual risk factor, we demonstrate what eGFR would be if an individual had a particular risk factor either independently or in conjunction with other risk factors. The numbers on the left side of the horizontal bars indicate the sample size, whereas the numbers to the right of each horizontal bar represent the eGFR for that condition. Overall eGFR is the average eGFR for individuals with one, two, or three risk factors; BMI = 1 indicates presence of obesity; HTN = 1 indicates hypertension; HDL = 1 indicates dyslipidemia as determined by the high-density lipoprotein variable; TG = 1 indicates dyslipidemia as determined by fasting triglycerides; and FG = 1 indicates high fasting glucose. The eGFR for the reference group (0 risk factors) is 103.93 mL/min/1.73 m^2^.

## 4. Discussion

The purpose of the present study was to report the prevalence of the strict metabolic phenotypes, renal function in each phenotype, and the risk factors associated with renal function. Our primary outcomes indicate that the strictly defined MUN phenotype accounted for the largest proportion of the U.S. population, whereas the MHO phenotype accounted for the smallest. In previous studies using the same strict definition of metabolic health, the MUN phenotype varied from 35–45% of the population, and the MHO phenotype ranged from 2.5–5.5% of the population, on average [11,12,23,34]. In the present study, the proportions of the “intriguing” phenotypes fall within the purviews of prior research. Similar overall results can be seen in previous studies [12,34] although the MHN and MUN populations can vary widely depending on the population measured. Kouvari et al. reported a large percentage of the MHN phenotype (36.30%) in the relatively homogenous Greek population assessed in the ATTICA cohort study [11], which is almost double the frequency of the MHN in the present study. Our prior research identified a large percentage of the MUO phenotype (57.79%) in a federally qualified health center in the southern U.S. [23], which is 1.5 times the proportion that we established here. The sample used in the present study is representative of the entire U.S. population and therefore consists of greater racial and ethnic diversity than the study by Kouvai et al. as well as greater socioeconomic and geographic diversity than our prior study.

In the study sample, renal function was lowest in the MUN phenotype. However, it is important to note that CKD was more prevalent in the MUO phenotype due to the definition of CKD and the high ACR ($A=\pi r^{2}$ = 44.45 mg/g, SE = 6.20) in the MUO phenotype. These findings persisted across multiple definitions of metabolic health ranging from the strict definition to the standard definition of MetS, demonstrating that one metabolic risk factor may be similarly indicative of renal dysfunction as two or three risk factors but that CKD status was more highly dependent upon ACR than eGFR. The MUN phenotype, while not typically perceived as high risk [10], has been correlated with adverse health outcomes, such as poor renal function [23], type 2 diabetes, cardiovascular events, and mortality [35]. In our study as well as previously reported findings [17,23], the MUN phenotype was correlated with older age. CKD has also been reported to be more common in individuals of older age [7], though this finding may be due to the prolonged presence of metabolic risk factors rather than age itself. A recent pilot study by Valdez et al. demonstrated that renal health was independent of age in individuals with no metabolic risk factors [36]. Still, more research is warranted to assess the renal risk in individuals with one or more metabolic risk factors and normal weight given that this constitutes a majority of the U.S. population.

Overweight (25 ≤ BMI ≤ 30) and obese (BMI ≥ 30) individuals have a 40% to 80% increased risk of CKD, respectively [37]. However, in the present study, the MHO phenotype presented with renal function that was comparable to the reference group (MHN). Similar to previous findings [38], the MHO phenotype was younger in age, indicating that the findings could be attributed to the short amount of time that these individuals have been in an obese state. In the early stages of obesity, the kidneys engage in compensatory vasodilation and hyperfiltration in an attempt to maintain sodium balance despite increased tubular sodium reabsorption [39]. Over time, the high-pressure system caused by hyperfiltration causes glomerulosclerosis, which may not be detectable via changes in serum creatinine values until renal function has decreased by approximately 50% [40]. The higher eGFR demonstrated in the MHO phenotype presents a phenomenon that may be explained by the transient state of “healthy obesity” wherein the detrimental metabolic effects of the obese state have not yet had time to manifest [11]. This finding demonstrates the inadequacy of BMI as a proxy measure for body composition, warranting future research on the relationship between body composition and renal function.

Long-term studies have demonstrated higher risk of CVD and mortality in the MHO phenotype [41,42]. Additionally, a longitudinal study by Kouvari et al. demonstrated that 52% of individuals classified as MHO transitioned to the MUO status within a 10-year timeframe [11]. While we cannot determine chronicity of disease in the present cross-sectional sample, we did observe possible indicators of future disease. A high hs-CRP level was detected in the obese phenotypes, which is indicative of systemic inflammation likely due to excess adipose tissue [43,44]. Additionally, the lipid profile of the MHO phenotype was within normal range yet inferior to that of the reference group. On average, triglycerides and LDL were 10 points higher than the MHN phenotype, and HDL was 5 points lower, increasing the risk of future CKD [45,46]. Although individuals classified as MHO have healthy metabolic and renal markers in cross-sectional analyses [47], it is likely that the inflammatory process of persistent obesity will be followed by metabolic risk factors and eventual declines in renal health. Further research is warranted to investigate the specific conditions necessary to maintain metabolic health in the presence of obesity.

In the overall sample, we found HTN, a high WC, and high fasting glucose to be negatively correlated with eGFR, which is intuitive given that hypertension and hyperglycemia are the two main precursors of CKD in the developed world [7]. In the MUN phenotype, eGFR had the largest correlations with HTN and WC. While these individuals were not obese as classified by BMI cutoffs, they did demonstrate a WC that was approximately 10 cm greater than that of the MHN, indicating that they carry more of their weight in the central region of their body. Central adiposity in the form of visceral adipose tissue (VAT) has been identified as a major contributor of insulin resistance [26] and is more metabolically active than subcutaneous fat or adipose tissue carried in the lower limbs [48]. The metabolically active VAT is possibly a major contributor to the metabolically unhealthy status and reduced renal function observed in this phenotype.

Unique to our study, HDL had a small negative association with eGFR, indicating that lower levels of HDL were correlated with a higher eGFR. This was demonstrated in Figure 1, where individuals with low HDL as one of their risk factors had a higher average eGFR than individuals with any other risk factor. The Pearson’s correlation analyses were consistent with these findings except for the MUN group, which displayed conflicting findings—indicating that eGFR increased as HDL decreased, but SCr increased while HDL decreased. The findings that higher HDL levels may increase risk of CKD contradict many previous research findings [38,45,46,49], but there have also been studies that confirmed greater risk of mortality associated with high HDL levels [50]. It is possible that the weak negative correlation demonstrated in our study could be explained by outliers with rare genetic variations in HDL receptors [51] or high levels of inflammation [52]. In future investigations, HDL function may prove to be more important than quantity. Still, further research should be done to understand these findings.

### Strengths and Limitations

This study is the first to utilize a strict definition of metabolic health in the assessment of CKD while also utilizing NHANES complex survey sample weighting techniques. Much of the research in metabolic phenotypes and renal function is conducted in Asian populations, whereas our sample was taken from a racially and ethnically diverse population in the U.S. The large sample size and use of the complex survey sample weighting techniques allowed us to report unique findings that are representative of the U.S. population. This study was limited by the cross-sectional nature of the data, which prevents us from making inferences about the temporal sequence of events leading to declines in renal function. NHANES sampling techniques and measures are widely accepted, yet selection bias may still occur. For example, 18 individuals who met the inclusion criteria of the present study reported dialysis in the past year. Given the voluntary nature of research, it is likely that few of the ill and/or infirmed individuals selected for this study chose to participate. To marginally correct for this, the sample weighting procedures adjust for nonresponse to reduce potential bias. The sample sizes of the four phenotypes varied widely, and the MHO phenotype was very small (5.59% of the population), lowering the statistical power in comparisons made using this phenotype. Additionally, the amount of variance explained by each of the regression models, demonstrated by the R2 values, was very low. A larger amount of variance could be explained by including variables such as age, sex, and race/ethnicity, but these values were considered in the equation estimating GFR and therefore were not added to the regression models. Metabolic risk factors, drug information, and BMI were considered in the metabolic phenotypes and therefore were not added to the regression equation. SES and caloric intake were not statistically different among the four phenotypes, and there was a large percentage of missingness in the PA, smoking, and hs-CRP variables, excluding these variables from the regression analyses. Therefore, the regression models are presented with the unadjusted results, which explains a small percentage of the variance in renal function yet demonstrates significant differences between the phenotypes. Glomerular filtration rate was estimated using an equation that utilizes serum creatinine, which can be affected by muscle mass, muscle breakdown, exercise, nutrition, medications, and hydration status. We were limited to one-time measures of eGFR and hs-CRP due to the cross-sectional nature of the study. To diagnose CKD, measures of SCr should be taken twice, approximately three months apart. Measures of hs-CRP should also be taken twice, approximately two weeks apart, to obtain an average measure of inflammation.

## 5. Conclusions

In the present study, we utilized a complex survey sample weighting technique to identify a sizable frequency of individuals with metabolic risk factors and/or obesity in the U.S. population. We observed higher proportions of males and individuals of older age in the metabolically unhealthy phenotypes, whereas in the obese phenotypes, we observed higher proportions of non-Hispanic Black individuals and greater levels of inflammation represented by hs-CRP values above 3.0 mg/L. Using a strict definition of metabolic health, we found that renal function was lowest in the MUN phenotype. These findings persisted when using more lenient definitions of metabolic health. The renal health of the MHO phenotype was not statistically different from the reference group; however, these findings are likely transient given previous reports from longitudinal studies. Hypertension, waist circumference, and HDL were negatively correlated with renal function, implicating future research in the area of dyslipidemia and renal function.

## Figures and Tables

**Table 1:** Criteria for metabolic risk factors and metabolic phenotypes.

Category	Classification	Values
**Metabolic Risk Factor**	Obesity	Non-Asian BMI ≥ 30 kg/m^2^, Asian BMI ≥ 25 kg/m^2^
	Hyperglycemia	Fasting glucose ≥100 mg/dL or Rx
	Dyslipidemia (2nd criteria)	TG ≥ 150 mg/dL or Rx
		HDL < 40 mg/dL (M), <50 mg/dL (F); or Rx
	Hypertension	>130 mmHg systolic or >85 mmHg diastolic or Rx
**Metabolic** **Phenotype**	MHN	Non-obese and <1 metabolic risk factor
	MHO	Obese and <1 metabolic risk factor
	MUN	Non-obese and >1 metabolic risk factor
	MUO	Obese and >1 metabolic risk factor

Metabolic syndrome is defined by the NCEP ATP III (2005 Revision) guidelines [26]. BMI is calculated as weight (kg) divided by height (m^2^). Rx, prescription medication for given risk factor; TG, triglycerides; HDL, high density lipoprotein; M, males; F, females; MHN, metabolically healthy normal weight; MHO, metabolically healthy obese; MUN, metabolically unhealthy normal weight; MUO, metabolically unhealthy obese.

**Table 2:** Demographic information for subsample from the 2013–2018 National Health and Nutrition Examination Survey.

	Unweighted Total (*n* = 6610)	Weighted Total (*n* = 220,388,819)	MHN (19.11%)	MHO (5.59%)	MUN (38.40%)	MUO (36.90%)	*p*-Value
	Mean (SD)	Mean (SE)	Mean (SE)	Mean (SE)	Mean (SE)	Mean (SE)	
Age (years)	47.03 (17.04)	45.61 (0.37)	35.72 (0.58)	36.22 (0.86)	49.31 (0.57)	48.31 (0.52)	<0.001
BMI (kg/m^2^)	29.4 (7.33)	29.40 (0.18)	23.42 (0.13)	33.48 (0.28)	25.33 (0.09)	36.10 (0.24)	<0.001
Waist Circumference (cm)	99.35 (17.15)	99.83 (0.43)	83.75 (0.40)	105.93 (0.84)	92.25 (0.30)	115.19 (0.50)	<0.001
Caloric Intake (Kcal/day)	2048 (853)	2087 (17)	2083 (43)	2032 (56)	2123 (25)	2058 (27)	0.207
Fasting Glucose (mg/dL)	110.71 (37.50)	107.74 (0.49)	91.54 (0.26)	92.41 (0.38)	108.35 (0.65)	117.83 (0.83)	<0.001
Triglycerides (mg/dL)	115.59 (112.38)	114.16 (1.70)	66.21 (1.21)	75.35 (1.81)	117.73 (2.03)	141.14 (3.20)	<0.001
HDL (mg/dL)	53.75 (16.11)	54.29 (0.36)	64.82 (0.65)	59.06 (1.07)	54.49 (0.53)	47.90 (0.37)	<0.001
LDL (mg/dL)	111.22 (35.56)	111.38 (0.72)	100.91 (1.32)	109.55 (1.79)	113.90 (1.13)	114.55 (1.01)	<0.001
Systolic BP (mmHg)	123.31 (18.00)	121.41 (0.29)	110.05 (0.40)	113.55 (0.49)	122.64 (0.44)	127.19 (0.37)	<0.001
Diastolic BP (mmHg)	70.13 (12.28)	70.30 (0.29)	65.32 (0.32)	68.11 (0.62)	70.49 (0.42)	73.01 (0.33)	<0.001
eGFR (mL/min/1.73 m^2^)	97.7 (22.17)	97.16 (0.50)	103.93 (0.91)	106.44 (1.25)	94.34 (0.64)	95.19 (0.61)	<0.001
hs-CRP (mg/L)	4.15 (8.25)	3.80 (0.18)	1.39 (0.07)	4.49 (0.52)	2.92 (0.26)	5.68 (0.28)	<0.001
ACR (mg/g)	41.66 (291.46)	29.14 (2.78)	16.70 (2.33)	10.49 (3.07)	23.34 (2.93)	44.45 (6.20)	<0.001
HOMA-IR	4.22 (8.52)	3.77 (0.10)	1.35 (0.03)	2.51 (0.10)	2.63 (0.07)	6.43 (0.22)	<0.001
SCr (mg/dL)	0.86 (0.28)	0.86 (0.00)	0.83 (0.00)	0.84 (0.01)	0.86 (0.00)	0.87 (0.01)	<0.001
BUN (mg/dL)	13.74 (5.24)	13.84 (0.12)	13.03 (0.16)	12.61 (0.31)	14.16 (0.18)	14.12 (0.16)	<0.001
	n (%)	% (SE)	% (SE)	% (SE)	% (SE)	% (SE)	*p*-value
Male Sex	3205 (48.49)	49.39 (0.67)	40.14 (2.28)	36.76 (3.24)	56.72 (1.48)	47.01 (1.44)	<0.001
Race/Ethnicity							
Mexican American	1041 (15.75)	9.49 (1.12)	8.02 (1.09)	8.97 (2.29)	9.06 (1.10)	10.78 (1.30)	<0.001
Other Hispanic	731 (11.06)	6.49 (0.79)	6.87 (1.34)	7.29 (1.75)	6.99 (0.85)	5.66 (0.65)	
NH White	2353 (35.60)	63.36 (1.98)	68.02 (2.66)	48.17 (4.57)	67.21 (1.89)	59.23 (2.44)	
NH Black	1376 (20.82)	11.29 (1.11)	6.77 (0.98)	30.32 (3.53)	3.54 (0.52)	18.81 (1.89)	
NH Asian	849 (12.84)	5.55 (0.52)	7.45 (0.76)	2.15 (0.63)	8.97 (0.97)	1.52 (0.16)	
Other/Multi-Racial	260 (3.93)	3.83 (0.40)	2.88 (0.58)	3.10 (1.01)	4.24 (0.59)	4.00 (0.61)	
Low SES	1355 (22.69)	15.43 (1.05)	13.10 (1.46)	16.56 (1.99)	15.22 (1.23)	16.67 (1.47)	0.143
CKD	966 (14.61)	12.07 (0.52)	6.31 (1.05)	3.60 (1.05)	11.73 (0.77)	16.70 (0.83)	<0.001
Physically Active	2317 (69.98)	69.38 (1.08)	77.93 (1.83)	76.24 (3.30)	67.79 (1.98)	62.88 (2.03)	<0.001
Smoker	2981 (45.10)	46.28 (1.26)	37.38 (2.46)	40.78 (3.55)	49.76 (1.57)	48.09 (1.43)	<0.001
Glucose Medication	797 (12.06)	9.14 (0.53)	0	0	8.57 (0.76)	15.86 (1.00)	<0.001
Cholesterol Medication	1206 (18.25)	17.27 (0.67)	0	0	21.64 (1.20)	24.29 (1.31)	0.158
Hypertension Medication	1678 (25.39)	22.09 (0.88)	0	0	23.23 (1.47)	35.69 (1.51)	<0.001

Metabolically healthy status is defined as having 0 risk factors, with the exception of obesity. *p*-values indicate a significant difference between the four metabolic phenotypes for the given variable. BMI, body mass index; HDL, high-density lipoprotein; LDL, low-density lipoprotein; BP, blood pressure; eGFR, estimated glomerular filtration rate using the CKDEPI equation; hs-CRP, high-sensitivity C-reactive protein; ACR, albumin to creatinine ratio; HOMA-IR, homeostatic model assessment for insulin resistance; SCr, serum creatinine; BUN, blood urea nitrogen; NH, Non-Hispanic; SES, socioeconomic status; CKD, chronic kidney disease, determined by eGFR < 60 and/or ACR ≥ 30. There was 18% missingness in the Caloric Intake variable, 49% in the Physically Active variable, and 36% missingness in the hs-CRP variable.

**Table 3:** Linear Regression Analyses.

*Coefficient*	Model 1 ^a^		Model 2 ^b^		Model 3 ^c^	
	*B*	*SE B*	*B*	*SE B*	*B*	*SE B*
*Intercept (MHN)*	*103.93*	*0.91*	*101.98*	*0.79*	*99.43*	*0.73*
MHO	2.50	1.42	2.03	1.15	1.54	0.80
MUN	−9.60 **	0.80	−12.30 **	1.09	−12.33 **	1.20
MUO	−8.74 **	0.96	−9.55 **	1.01	−10.53 **	0.96
R^2^	0.042		0.077		0.059	

** *p* < 0.001. ^a^ Metabolic health defined as 0 metabolic abnormalities (with the exception of obesity) and 1 risk factor considered unhealthy. ^b^ Metabolic health defined as 1 metabolic abnormality (with the exception of obesity) and 2 risk factors considered unhealthy. ^c^ Metabolic health defined as 2 metabolic abnormalities (with the exception of obesity) and 3 risk factors considered unhealthy.

**Table 4:** Correlates of eGFR.

	Overall eGFR	Overall SCr	MHO eGFR	MHO SCr	MUN eGFR	MUN SCr
FG, *r*	−0.119 **	0.026 *	0.015	−0.023	−0.069 **	−0.020
n	6610	6588	367	366	2537	2529
TG, *r*	−0.083 **	0.040 *	−0.159 *	0.042	−0.044 *	0.034
n	6610	6588	367	366	2537	2529
HDL, *r*	−0.002	−0.123 **	−0.065	−0.172 *	−0.088 **	−0.126 **
n	6610	6588	367	366	2537	2529
SBP, *r*	−0.25 **	0.105 **	0.008	0.078	−0.269 **	0.106 **
n	6610	6588	367	366	2537	2529
DPB, *r*	−0.023	0.011	−0.084	−0.067	0.00	0.020
n	6610	6588	367	366	2537	2529
BMI, *r*	−0.056 **	0.011	0.049	−0.165 *	−0.124 **	0.061 *
n	6610	6588	367	366	2537	2529
WC, *r*	−0.175 **	0.096 **	−0.033	−0.123 *	−0.282 **	0.187 **
n	6445	6424	358	357	2481	2473

* *p* < 0.05, ** *p* < 0.001; SCr, serum creatinine; *r*, Pearson’s Correlation Coefficient; n, number of observations; eGFR, estimated glomerular filtration rate; FG, fasting glucose; TG, triglycerides; HDL, high-density lipoprotein; SBP, systolic blood pressure; DBP, diastolic blood pressure; BMI, body mass index; WC, waist circumference.

## Data Availability

All data are available from the CDC at https://wwwn.cdc.gov/nchs/nhanes/.
